# Work Burnout and Engagement Profiles Among Teachers

**DOI:** 10.3389/fpsyg.2019.02254

**Published:** 2019-10-04

**Authors:** Katariina Salmela-Aro, Lauri Hietajärvi, Kirsti Lonka

**Affiliations:** ^1^Faculty of Educational Sciences, University of Helsinki, Helsinki, Finland; ^2^ETH Zurich, Zurich, Switzerland; ^3^Optentia Research Focus Area, North-West University, Vanderbijlpark, South Africa

**Keywords:** teachers, work engagement, burnout, person-oriented approach, resilience

## Abstract

The focus of the current study was to examine teachers’ well-being in terms of work engagement and burnout by using a person-oriented approach. The participants (*n* = 149, 70.5% female) were subject-matter teachers from 22 schools from metropolitan Helsinki area in Finland. The first aim was to examine the kinds of profiles we can identify based on work burnout and engagement among teachers. The second aim was to study how the identified profiles differed in job-related demands and resources and personal resources in terms of resilience. Based on the demands-resources model, we expected to find profiles that differ in terms of key resources and demands. The sample was acquired as a convenience sample and the data was collected using online self-report questionnaires. The measures were work engagement, work burnout, work demands/resources (workload and control) and resilience as the personal resource. In addition, changes and effects of the economic circumstances were accounted for with two binary variables assessing the effect on class sizes and material resources. We identified two profiles among teachers: engaged (30%) and engaged-burnout (70%) profiles. We found that those in the engaged profile group had more job and personal resources, such as control and resilience, whereas those in the engaged-burnout profile group experienced more work demands, such as workload.

## Introduction

Teacher burnout has been identified as a worldwide problem (Tsouloupas et al., 2010; Dicke et al., 2015, 2017). Studies have focused on teachers’ burnout, stress and high attrition, and, thus a lack of teachers. Finland is a rare country in the sense that teacher profession is highly appreciated and wanted profession so far; it has been difficult to get in to teacher education programs that are at the level of Master degree, the requirement of working as a teacher (Lonka, 2018). Still, teachers have been found two decades ago to suffer from burnout levels when compared to workers in other white collar jobs (Kalimo and Hakanen, 2000). This is very alarming as teachers wellbeing is related to students’ wellbeing (Koh et al., 1995; Day, 2011; Klusmann et al., 2016). Thus there is a need to identify the amount of teachers suffering from burnout. Moreover, we have to identify the risk as well as protective factors for developing burnout symptoms among teachers.

However, we have to be aware that the majority of teachers are engaged about their work (Kinnunen et al., 1994). In Finland, teachers have high autonomy and control over their work and there are no inspections or yearly standardized tests to monitor teachers (Sahlberg, 2010). On the other hand, a new national curriculum is introduced every 10 years and the profession is constantly under reforms and profound changes. Even though the teachers’ high competence, their working conditions are becoming more challenging as increasingly heterogeneous pupil population due to immigration and reforms toward inclusive special needs education, endeavors to digitalize schools, as well as cuts and savings in school funding. The profession is thus simultaneously likely to promote both engagement and burnout because of constant new challenges and requirements. Thus, our aim was to take a person-oriented approach and examine simultaneous work engagement and burnout profiles among teachers and their antecedents and consequences in the context of demands-resources model. Under such working conditions is possible to find teachers who are highly engaged, but still may report exhaustion or other symptoms of burnout.

In person-oriented approach compared to variable-oriented approach it is possible to find different profiles of participants and there is still very few studies with regard to occupational wellbeing for educational personnel. A key benefit of a person-oriented approach is the possibility to identify the participants endorsing seemingly contradictory measures of work well-being, such as burnout and engagement, simultaneously (Moeller et al., 2018). Latent profile analysis brings the capacity to guide identifying the ideal number of profiles in burnout and engagement (see also Leiter and Maslach, 2016). However, there is, in particular, lack of studies to examine teachers’ simultaneous burnout and engagement profiles. Some studies have identified teacher profiles (Klusmann et al., 2008; Collie et al., 2015) but none of the previous studies have examined work burnout and engagement profiles among teachers. Previous studies in educational context, among elementary (Salmela-Aro et al., 2017), high school (Tuominen-Soini and Salmela-Aro, 2014) and higher education (Salmela-Aro and Read, 2016) students rather than teachers have identified three to five profiles, engaged, engaged-burnout and burnout profiles. Moreover, previous studies among occupational context in general have identified mostly two profiles, engaged and burnout (Innanen et al., 2015). Based on the previous studies we expected to identify two to three profiles among teachers, engaged, engaged-burnout and possibly even burnout profiles. Based on the professional profile, it was expected that exhaustion would be more common than cynicism, because teachers have good chances for experiencing autonomy, competence and relatedness that support autonomous and intrinsic motivation as well as personal engagement (Deci and Ryan, 2008; Skinner et al., 2014; Rothmann and Cooper, 2015).

The Job Demands-Resources (JD-R) model was used to examine job burnout and engagement profiles (Demerouti et al., 2001; Bakker et al., 2003a; Bakker and Demerouti, 2006). Job demands-resources model proposes a motivational process from job resources to work engagement and an energetic process from demands to job burnout. In the present study, we integrate the energetic and motivational processes by examining simultaneous burnout and engagement profiles among teachers (Hakanen et al., 2006; Upadyaya et al., 2016). Work burnout often reflects employees’ reaction to chronic occupational stress, and work burnout has been conceptualized in terms of inadequacy, exhaustion, and cynicism at work (Maslach et al., 2001; Schaufeli et al., 2002). Exhaustion refers to strain and overtaxing work (González-Romá et al., 2006). Cynicism refers to negative work attitude, disinterest and low work meaning (Salmela-Aro et al., 2009). Finally, inadequacy refers to inadequacy at work and decreased accomplishments (Schaufeli et al., 2002). These refer to emotional, cognitive, and behavioral components of burnout (Salmela-Aro and Upadyaya, 2014). In turn, work engagement can be defined as high dedication, energy, and absorption at work (Schaufeli et al., 2002). Resilience and vigor characterizes the energy component (Schaufeli et al., 2002). Meaning, pride, and inspiration refer to work dedication (Schaufeli et al., 2002). Finally, absorption is similar to flow so that time passes quickly at work (Schaufeli et al., 2002). These refer to emotional, cognitive and behavioral components of work engagement (Salmela-Aro and Upadyaya, 2014).

According to the JD-R model (Demerouti et al., 2001; Bakker et al., 2003a) high level of psychological, social, physical, and organizational demands are related to work burnout, whereas psychological, social, physical, and organizational resources are related to work engagement (Xanthopoulou et al., 2009; Alarcon, 2011; Salmela-Aro and Upadyaya, 2014). Key identified work demands are workload, time pressure, and long working hours (Bakker et al., 2003b) but also interpersonal conflicts (Ilies et al., 2015). These have been found to increase teachers’ burnout (Pietarinen et al., 2013). Key job-related resources refer to positive team climate, and control. In turn, personal resources refer to employees’ self-efficacy, self-esteem, and resilience, and they are associated to work engagement (Bakker and Demerouti, 2008; Salmela-Aro and Upadyaya, 2014). Consequently, work and personal resources can reduce work demands as they help to achieve work-related goals (Demerouti and Bakker, 2011). It has also been found that personal resources may act as a buffer so that the possible negative association from work demands to work burnout is weaker (Hakanen et al., 2006; Bakker et al., 2007; Upadyaya et al., 2016; Spini et al., 2017). Recent studies have revealed that resilience is an important personal resource, as it can act as a protective factor that allows teachers to react using adaptive coping when they face challenges and problems in their work (Skinner et al., 2014). In the present study we focused on the key job demands which might be particularly important among teachers, such as workload and large class size, and job resources which might be particularly important as major motivations in teachers job that increase work engagement, or when lacking increase work burnout, such as control and material resources. Finally, personal resources can buffer and lead to work engagement. The present study included resilience as a personal resource.

The aim of the present study was to identify profiles among teachers based on work burnout and engagement. Based on the previous studies we expected to identify two to three profiles among teachers, engaged, engaged-burnout, and burnout profiles (H1). The second aim was to analyze if the identified profiles differ in job-and personal resources and job demands. Based on the demands-resources model, we expected to profiles differ in terms of resources and demands (H2). We expected those in more engaged profile group to have more job and personal resources, such as control and resilience, whereas those in the burnout profile group to have more work demands, such as workload than those in the more engaged profile group.

## Materials and Methods

### Participants

The participants were 149 (70.5% female) subject matter teachers from 22 comprehensive and high schools from Metropolitan Helsinki area. The sample was acquired as a convenience sample and the data was collected using online self-report questionnaires. Out of the participants 76% were from high schools, 15.1% from schools that provide both comprehensive and high school education and 8.2% were from comprehensive schools. The average age of the participants were 45.62 years (*SD* = 8.56) and the average experience as a teacher was 16.93 years (*SD* = 8.76). Participation in the study was voluntary and informed consent were acquired as a part of the questionnaire. The study protocol was approved by the University of Helsinki Ethical Review Board in the Humanities and Social and Behavioral Sciences.

### Measures

#### Work Engagement

Work Engagement was measured with a short version of the Utrecht Work Engagement Scale, UWES-S (Schaufeli et al., 2006) which consists of nine items measuring work-related energy (“When I work, I feel that I am bursting with energy”), dedication (“I am enthusiastic about my work”), and absorption (“Time flies when I’m working”) to be rated on a 7-point scale (0 = not at all to 6 = daily). These items refer to emotional, cognitive and behavioral aspects of work engagement. We used the overall measure of work engagement (Schaufeli et al., 2006). A sum score was formed to measure the employees’ overall work engagement. The Cronbach’s alpha reliability for the scale was 0.90.

#### Work Burnout

Work Burnout was measured with the Bergen Burnout Inventory (Salmela-Aro et al., 2004; see also Näätänen et al., 2003; Salmela-Aro et al., 2011) which consists of 9 items measuring three factors of job burnout: exhaustion (“I feel overwhelmed by my work”, α = 0.73); cynicism about the work meaning of work (“I feel lack of motivation in my work and often think of giving up”, α = 0.79), and inadequacy (“I often have feelings of inadequacy in my work”, α = 0.80) rated on a 6-point scale (1 = strongly disagree to 6 = strongly agree). These refer to emotional, cognitive and behavioral aspects of work burnout. Sum scores were formed to represent the different components of burnout.

#### Work Demands and Resources

Work demands was measured by workload and work resources as control. They were measured with items taken from the Areas of Worklife Survey (Leiter and Maslach, 2016). Workload consisted of 2 items (e.g., “I don’t have time for all the work that needs to be done.” α = 0.68). Control consisted of 2 items (e.g., “I have control over how I do my work”. α = 0.54). The items were rated on a 5-point likert-type scale (1 = completely disagree; 5 = completely agree).

#### Personal Resources

*Personal resources* were measured by resilience. Resilience was measured with 8 items (e.g., “When I encounter difficulties in my work, if usually find multiple solutions.” α = 0.90) to be rated on a 5-point likert scale (1 = completely disagree to 5 = completely agree) (Smith et al., 2008).

In addition, changes and effects of the economic circumstances were accounted for with two binary variables assessing the effect on class sizes (“Have the class sizes increased due to economic circumstances”) and the effect on material resources (“Have the material resources diminished due to economic circumstances and affected general academic performance”).

### Analysis Strategy

First, data was screened for possible outliers and missing values in SPSS 24^1^. There were 0.62% of values missing. Little’s MCAR test showed that these were missing completely at random (Chi-Square = 139.107, *DF* = 156, Sig. = 0.830). No outliers were identified. Further analysis were conducted with R version 3.5.3 and RStudio (R Core Team, 2015, 2018). The sum scores were computed with the restriction that 50% of the items in each scale were required to have a valid value in order to be computed, otherwise the sum variable were coded missing (package “sjstats”, Lüdecke, 2018). As a measure of internal consistencies there were estimated Cronbach’s Alphas (package “MBESS”, Kelley, 2018). See Table 1 for descriptive values of the variables.

**TABLE 1:** Variable descriptives.

										**Cronbach’s**				
**Variable**	***n***	**Range**	***M***	***SD***	**SE**	**Min**	**Max**	**Skew**	**Kurtosis**	**Alpha**	**SE**	**95% C.I.^∗^**		
Engagement	149	1 to 7	6.06	0.82	0.07	2.22	7.00	–1.53	3.66	0.88	0.03	0.83	–	0.93
Exhaustion	148	1 to 6	3.03	1.21	0.10	1.00	6.00	0.28	–0.50	0.73	0.04	0.63	–	0.80
Cynicism	148	1 to 6	2.03	1.07	0.09	1.00	6.00	1.30	1.63	0.79	0.05	0.67	–	0.86
Inadequacy	148	1 to 6	2.35	1.32	0.11	1.00	6.00	0.77	–0.57	0.80	0.03	0.73	–	0.85
Workload	148	1 to 5	2.98	1.06	0.09	1.00	5.00	–0.03	–0.69	0.68	0.06	0.55	–	0.77
Control	148	1 to 5	3.62	0.85	0.07	1.00	5.00	–0.66	0.60	0.54	0.09	0.34	–	0.68
Resilience	149	1 to 5	4.09	0.60	0.05	2.43	5.00	–0.40	–0.41	0.90	0.01	0.86	–	0.92
Class size	149	0 to 1	0.82	0.39	0.03	0.00	1.00	–1.64	0.69	–	–	–		–
Material resources	147	0 to 1	0.71	0.45	0.04	0.00	1.00	–0.94	–1.13	–	–	–		–

*^∗^Bias-corrected and accelerated bootstrap with 10,000 draws.*

Latent profile analyses (see e.g., Vermunt and Magidson, 2002) were conducted to identify latent subgroups of teachers regarding their work engagement and burnout. Analyses were conducted with “tidyLPA” package in R including a simple single imputation and standardization to the variables prior to model estimation as implemented in the package (Rosenberg et al., 2019). To estimate the best fitting model we relied most on Bayesian information criterion (BIC), which has shown to be robust across a variety of conditions (Nylund et al., 2007). Subsequent binomial logistic regressions were conducted to examine the relations of covariates and the profile membership, the statistical significance of the effects were evaluated with the conventional alpha level of *p* < 0.05 with, however, confidence intervals also presented.

## Results

First, we compared solutions with different restrictions for variances and covariances between different profiles (for details see Table 2). This was done to identify the model that would fit our data best. With each specification we estimated models with increasing number of profiles. All models except Model 1 (see Table 2) suggested that a two-profile solution would be best and out of these the lowest BIC was estimated with the Model 4 specification in which means, variances and covariances were allowed to be freely estimated across profiles. As the two profile solution was also substantively meaningful we decided to proceed with the two profile solution.

**TABLE 2:** Model fit indices of latent profile analyses for all models compared.

					**Prob**	**Prob**			**BLRT**
**Model**	**Classes**	**AIC**	**BIC**	**Entropy**	**min**	**max**	**% min**	**% max**	***p*-value**
1 Equal variances and covariances fixed to 0	1	1802.01	1826.04	1.00	1.00	1.00	1.00	1.00	
1 Equal variances and covariances fixed to 0	2	1616.15	1655.20	0.89	0.94	0.98	0.28	0.73	0.01
1 Equal variances and covariances fixed to 0	3	1557.60	1611.67	0.90	0.93	0.98	0.14	0.57	0.01
2 Varying variances and covariances fixed to 0	1	1802.01	1826.04	1.00	1.00	1.00	1.00	1.00	
2 Varying variances and covariances fixed to 0	2	1503.57	1554.64	0.95	0.98	0.99	0.32	0.69	0.01
2 Varying variances and covariances fixed to 0	3	1496.52	1574.62	0.95	0.93	0.99	0.09	0.68	0.11
3 Equal variances and equal covariances	1	1586.00	1628.05	1.00	1.00	1.00	1.00	1.00	
3 Equal variances and equal covariances	2	1550.59	1607.66	0.85	0.89	0.98	0.26	0.75	0.01
3 Equal variances and equal covariances	3	1558.32	1630.42	0.65	0.64	0.89	0.19	0.56	0.89
**4** Varying variances and varying covariances	**1**	**1586.00**	**1628.05**	**1.00**	**1.00**	**1.00**	**1.00**	**1.00**	
**4** Varying variances and varying covariances	**2**	***1433.53***	***1520.65***	***0.92***	***0.98***	***0.99***	***0.30***	***0.70***	***0.01***
**4** Varying variances and varying covariances	**3**	**1418.48**	**1550.65**	**0.93**	**0.97**	**1.00**	**0.12**	**0.62**	**0.10**

*The model specifications refer to holding variances/covariances equal, fixed to 0 or freely estimated *between classes*. The selected model specification bolded and the selected model italicized.*

**FIGURE 1:**
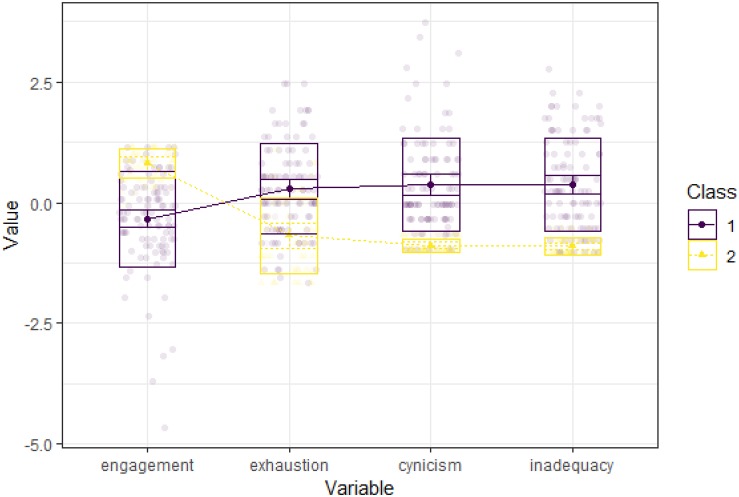
The two latent profiles of teacher work burnout and engagement. Profile 1: engaged-burnout (*n* = 104), Profile 2: highly engaged (*n* = 45).

The two profiles we identified represented two distinct types of teachers (see Figure 1 and Table 3): Profile 1 was the larger profile (70%) and consisted of teachers that were quite engaged, although slightly lower than the sample mean (*z* = −0.35, a small effect). They, however, also experienced more symptoms of burnout than the profile 2. In all burnout symptoms they were higher than the sample mean (*z* = 0.30 to 0.39, small effects). The profile was labeled as engaged-burnout. Profile 2 (30%) consisted of teachers that were more engaged (*z* = 0.80, a large effect) and experienced less symptoms of burnout (*z* = −0.70 to −0.91, large effects) than the profile 1. The profile was labeled as highly engaged. As the entropy of the selected solution was high (0.92) we saved the most likely profile memberships for subsequent analysis.

**TABLE 3:** Mean differences between profiles in indicator variables.

	**Engaged - burnout**			**Highly engaged**		
	**(*n* = 104)**			**(*n* = 45)**		
	***M***	***SD***	***z***	***M***	***SD***	***z***
Engagement	5.77	0.82	–0.35	6.72	0.25	0.80
Exhaustion	3.39	1.12	0.30	2.19	0.96	–0.70
Cynicism	2.44	1.03	0.39	1.09	0.15	–0.89
Inadequacy	2.87	1.26	0.39	1.17	0.23	–0.91

**z*, standardized difference from sample mean.*

We then specified a binomial logistic general linear model in which we assigned the profile membership (profile 2 as compared to profile 1) as dependent variable and gender, workload, control, resilience and the two financial context measures as independent variables (see Table 4). Teachers with a higher workload and who reported increase in class sizes due to economic circumstances were more likely to belong to Profile 1 (engaged-burnout), whereas teachers who experienced more control over their work and reported higher resilience were more likely to be assigned to Profile 2 (highly engaged).

**TABLE 4:** Results of the binary logistic regression.

**Independent**					
**variables**	**Estimate**	**SE**	**z**	***p***	**95% C.I.^∗^**
(Intercept)	–15.89	3.59	–4.42	0.000	−22.16 to −7.62
Male	–1.48	0.70	–2.13	0.033	−2.99 to 0.01
Workload	–1.15	0.32	–3.55	0.000	−1.93 to −0.30
Control	1.68	0.48	3.54	0.000	0.70 to 2.66
Resilience	2.95	0.72	4.10	0.000	1.36 to 4.28
Class size	–1.31	0.75	–1.73	0.083	−3.28 to 0.47
Material resources	1.06	0.64	1.64	0.100	−0.54 to 2.49

*Estimates show the probability for belonging in profile 2 “Highly Engaged” as compared to profile 1. ^∗^Bias-corrected and accelerated bootstrap with 1000 draws.*

## Discussion

The results of the previous study among teachers showed that two profiles could be identified based on teachers’ burnout and engagement. These two profiles represented two distinct types of teachers and supported our hypothesis. The first profile was larger (70%) and consisted of teachers that were quite engaged but also experienced more symptoms of burnout than the other group. This group was named as engaged-burnout group. This group is in line with the earlier studies among students in high school which has identified a group of students who are engaged but have at the same time some symptoms of burnout (Salmela-Aro and Read, 2017). This is an important finding as this profile has not been previously identified among teachers. However, the signs of burnout were only emerging and more studies are needed to replicate the findings. In turn, the second profile was smaller (30%) and consisted of teachers that were very engaged and experienced low level of symptoms of burnout. This group was named as highly engaged group. These results show that over two times more teachers belong to the engaged-burnout group compared to the highly engaged group.

Interestingly, the person-oriented approach used in the present study showed that both groups were high in engagement. Teachers seem to be very committed and engaged to their work, whereas many suffer at the same time some signs of exhaustion and inadequacy as a teacher. This is a new but also a worrying result. Earlier studies among students have identified this kind of engaged-exhausted profile to be quite successful in the short–term but it has been found to lead to severe costs in the long run. Earlier studies have shown this group in particular to be risk of depressive symptoms later on (Tuominen-Soini and Salmela-Aro, 2014). Depression is one of the leading risks for early retirement and dropping out of teacher profession and thus these results need to be taken very seriously. This result may indicate that even highly educated, autonomous teachers who experience their work meaningful and who are engaged, may suffer of some symptoms of burnout. It is worth pointing out that, fortunately, cynicism was not very high even in this profile. Teachers value their work and see their work as meaningful.

Supporting the demands-resources model and our hypothesis 2, the results showed further that engaged-burnout and engaged group differed in terms of the key work-related demands and work- and personal resources (Bakker et al., 2003a; Hakanen et al., 2006). Teachers with a higher workload and who reported increase in class sizes due to economic circumstances were more likely to belong to engaged-burnout group, whereas teachers who experienced more control over their work and reported higher resilience were more likely to be assigned to engaged group. Even when facing high challenges and heavy workload, sense of meaning and resilience may help teachers to cope with their work without becoming cynical or feel inadequate. On the other hand, the results should put into a larger context: high workload and increase in class size are school-level problems that may really make teachers to lose control (or at least sense of control) over their own work. The fact that the majority of teachers in this study belonged to the engaged-burnout cluster indicates that the working conditions calls for serious attention by the policy makers and municipals.

## Limitations and Future Research

The present study has some limitations. The study was based on teachers self-reports, and the sample was quite small. The reliability of some variables was 0.60 and one even below (0.54). By conventional psychometric criteria, any values of coefficient alpha below 0.6 would be regarded as poor, even for relatively heterogeneous constructs that are not regarded as high-stake psychological tests or (Richardson, 2004). Only one Cronbach’s Alpha in our study was below this limit. There is always a trade-off between a wish to increase reliability and simultaneously, to restrict the length of an instrument, because reliability coefficients always become better as the test is prolonged (DeVellis, 1991). In our pilot studies it appeared that teachers were very similar to medical students in terms of not tolerating long series of questions (Lonka et al., 2008; Vedenpää and Lonka, 2014).

We therefore ended up with some scales with only two to three items. To maximize the number of participants, we created an instrument that was reasonably short and still satisfactory in terms of reliability.

In addition, the study was cross-sectional as a design. Future studies needs to be carried out with larger samples and with longitudinal design. Longitudinal design could reveal the possible long-term dark side of the engaged burnout group of teachers. During recent year, Finnish teachers working conditions have become ever so challenging: a new ambitious and innovative national curriculum was introduced in 2016 with increasingly inclusive school policy and fast digitalization. At the same time the Government cut funding from schools. Thus it is important to monitor the development of well-being with larger numbers of teachers.

Moreover, in the future studies we need to examine at the same time both teachers and students and to reveal possible spill over and buffering effects. In addition, besides teachers, there is a need to study also the principals as they play a key role for empowering and motivating teachers and the school as an organization. The role of principals’ servant leadership as a possible buffer for teacher burnout is an interesting future research question (Upadyaya et al., 2016). Intervention studies are strongly needed to identify burnout risk teachers and prevent them from burnout out and leaving the profession.

## Conclusion

The person-oriented study identified two profiles of teachers, engaged-burnout (70%) and highly engaged (30%) ones. The study revealed first time a profile of teachers being engaged but at the same time in a risk of exhaustion and inadequacy. Supporting demands-resources model both personal and work related resources we more typical for the highly engaged group, whereas the work-related demands were more typical among the engaged-burnout group. The results are important from both the perspective of fostering resilience among teachers, but also in looking at the policy issues in the larger context of schools and the educational system. We would also recommend new forms of teachers’ crafting options to develop their work in order to help them to keep up with the increasing demands of teacher work in the rapidly changing information society and increasingly heterogeneous student population (Upadyaya et al., 2016). Identifying risk factors for teacher burnout is important. Even those teachers who report to be engaged, but simultaneously also exhausted, may have a risk to develop burnout. It is crucial to invest in teachers’ well-being and working conditions. Even excellent teachers have their limits in terms of how much change they can tolerate at the same time. Cutting funds and introducing ambitious reforms at the same time may not be a good idea. Theoretically, approaching teacher engagement and burnout simultaneously, from the point of view of resources and demands, is important in quickly changing information society. It is important to see the risks on time, before the teachers would really burn out.

## Data Availability Statement

The datasets generated for this study are available on request to the corresponding author.

## Ethics Statement

The studies involving human participants were reviewed and approved by the University of Helsinki Ethical Review Board in the Humanities and Social and Behavioral Sciences. The patients/participants provided their written informed consent to participate in this study.

## Author Contributions

KS-A planned the design and analyses, and participated in the writing process. LH was responsible for the data analyses and reporting of the statistical results. KL organized the collection of the data as the Principal Investigator of the project and contributed in the writing of the manuscript.

## Conflict of Interest

The authors declare that the research was conducted in the absence of any commercial or financial relationships that could be construed as a potential conflict of interest.

## Footnotes

1 https://www.ibm.com/analytics/data-science/predictive-analytics/spss-statistical-software
